# Blood Biomarkers Variations across the Pre-Season and Interactions with Training Load: A Study in Professional Soccer Players

**DOI:** 10.3390/jcm10235576

**Published:** 2021-11-27

**Authors:** Filipe Manuel Clemente, Francisco Tomás González-Fernández, Halil Ibrahim Ceylan, Rui Silva, Saeid Younesi, Yung-Sheng Chen, Georgian Badicu, Paweł Wolański, Eugenia Murawska-Ciałowicz

**Affiliations:** 1 Escola Superior Desporto e Lazer, Instituto Politécnico de Viana do Castelo, Rua Escola Industrial e Comercial de Nun’Álvares, 4900-347 Viana do Castelo, Portugal; rui.s@ipvc.pt; 2 Instituto de Telecomunicações, Delegação da Covilhã, 1049-001 Lisboa, Portugal; 3 Department of Physical Activity and Sport Sciences, Pontifical University of Comillas, 07013 Palma, Spain; francis.gonzalez.fernandez@gmail.com; 4 SER Research Group, Pontifical University of Comillas, 07013 Palma, Spain; 5 Physical Education and Sports Teaching Department, Kazim Karabekir Faculty of Education, Ataturk University, Erzurum 25240, Turkey; halil.ibrahimceylan60@gmail.com; 6 Research Unit for Sport and Physical Activity, Faculty of Sport Sciences and Physical Education, University of Coimbra, 3004-531 Coimbra, Portugal; saeidyounesi78@yahoo.com; 7 Department of Exercise and Health Sciences, University of Taipei, Taipei 11153, Taiwan; yschen@utaipei.edu.tw; 8 Department of Physical Education and Special Motricity, University Transilvania of Brasov, 500068 Brasov, Romania; georgian.badicu@unitbv.ro; 9 Department of Physiology, Gdansk University of Physical Education and Sport, 80-336 Gdansk, Poland; pawel.wolanski@awf.gda.pl; 10 Department of Physiology and Biochemistry, University School of Physical Education, 51-612 Wrocław, Poland; eugenia.murawska-cialowicz@awf.wroc.pl

**Keywords:** soccer, performance, biology, workload

## Abstract

**Simple Summary:**

Sports training may impact the variations of biomarkers in soccer players. Twenty-five professional soccer players were assessed twice in the season for their hematology and biochemical status, while training loads were monitored over the season. Relationships between changes in biomarkers and accumulated training loads were tested. Results revealed that that intense training in the pre-season period leads to decreases and increases in different hematological and biochemical markers.

**Abstract:**

**Background:** Pre-season training in soccer can induce changes in biological markers in the circulation. However, relationships between chosen hematological and biochemical blood parameters and training load have not been measured. **Objective:** Analyze the blood measures changes and their relationships with training loads changes after pre-season training. **Methodology:** Twenty-five professional soccer players were assessed by training load measures (derived from rate of perceived exertion- known as RPE) during the pre-season period. Additionally, blood samples were collected for hematological and biochemical analyses. **Results:** For hematological parameters, significant increases were found for platelets (PLT) (dif: 6.42; *p* = 0.006; d = −0.36), while significant decreases were found for absolute neutrophils count (ANC) (dif: −3.98; *p* = 0.006; d = 0.11), and absolute monocytes count (AMC) (dif: −16.98; *p* = 0.001; d = 0.78) after the pre-season period. For biochemical parameters, there were significant increases in creatinine (dif: 5.15; *p* = 0.001; d = −0.46), alkaline phosphatase (ALP) (dif: 12.55; *p* = 0.001; d = −0.84), C-reactive protein (CRP) (dif: 15.15; *p* = 0.001; d = −0.67), cortisol (dif: 2.85; *p* = 0.001; d = −0.28), and testosterone (dif: 5.38; *p* = 0.001; d = −0.52), whereas there were significant decreases in calcium (dif: −1.31; *p* = 0.007; d =0.49) and calcium corrected (dif: −2.18; *p* = 0.015; d = 0.82) after the pre-season period. Moreover, the Hooper Index (dif: 13.22; *p* = 0.01; d = 0.78), and all derived RPE measures increased after pre-season period. Moderate-to-very large positive and negative correlations (*r* range: 0.50–0.73) were found between the training load and hematological measures percentage of changes. Moderate-to-large positive and negative correlations (*r* range: 0.50–0.60) were found between training load and biochemical measures percentage of changes. **Conclusions:** The results indicated heavy physical loads during the pre-season, leading to a decrease in immune functions. Given the significant relationships between blood and training load measures, monitoring hematological and biochemical measures allow coaches to minimize injury risk, overreaching, and overtraining.

## 1. Introduction

Elite soccer has intermittent characteristics that require players to frequently engage in a high level of aerobic and anaerobic capacity [1]. Average VO_2_max values achieved by soccer athletes can reach up to approximately 63 mL/kg/min. While, maximal aerobic speed (MAS) can reach up to 17 km/h [2]. Professional soccer players have to perform low-intensity activities interspersed with high-intensity short explosive actions during training and matches [3].

Indeed, modern soccer is characterized by increasingly demanding physical activities during both training sessions and matches [4]. In fact, professional players can cover up to 7000 m of total distances (TD) in a single training session, and approximately 13,000 m during a match [5]. From the above-mentioned TD volume, players are required to cover significant distances in different high-intensity velocity thresholds, such as high-intensity running (HIR), high-speed running (HSR), sprints, and accelerations and decelerations [6,7]. Furthermore, different positions in the field require different physical demands. Therefore, it is essential to consider not only the biological individuality of each player, but also the physical demands of each position on the field [8].

As mentioned above, the pre-season is considered a critical period as, overall, players need to improve their fitness levels after the offseason period [9]. The detraining effects of the offseason period are accompanied by impairments in both physical and skills performance, that may be more pronounced if there is no individualized training program during the offseason [9,10]. Despite that, a study conducted on 23 elite soccer players showed improvements of approximately 8% in their aerobic and anerobic performance after a pre-season period [11]. Furthermore, physical and physiological changes during the in-season can be dependent on the physical and physiological status observed at the beginning of the season [12]. However, a recent study showed that improvements in aerobic fitness after a pre-season period may not happen in a linear fashion as the authors found that fitness changes after the pre-season have a great variability between different seasons [13].

For such reasons, it is of paramount importance to monitor internal load measures on a daily basis. There are several psychometric measures, including fatigue, stress, soreness, quality of sleep factors, and their respective Hooper Index score (sum of the four factors), to monitor the well-being status of each player on a daily basis [14,15]. The Hooper Index score has been associated with the training load in soccer, showing its usefulness for practice [16]. In fact, a recent study conducted on nine professional soccer players revealed that the Hooper Index score had lower typical errors than the heart rate variability [17]. Thus, its usefulness seems to be promising in monitoring player’s fatigue during a soccer season. Furthermore, the load monitoring can be daily applied using subjective measures. Those measure are based on the rate of perceived exertion (RPE) scales to obtain an indicator of global internal load of soccer training sessions, such as the session-rate of perceived exertion (s-RPE) [18]. In addition, other authors have started to use other RPE measures in their investigations, such as the sRPE general, sRPE breath, and sRPE neuromuscular [19,20]. These new s-RPE measures can determine the subjective perception of exertion on different body structures [20]. However, Los Arcos et al. [21], revealed no relationships between sRPE general, sRPE breath, and sRPE neuromuscular with changes in aerobic fitness.

Besides the common influencers of aerobic fitness (e.g., ventilatory kinetics, cardiac process, neuromuscular status), other hematological and biochemical parameters assume a preponderant role in athletes’ performance [22]. However, there is incongruent evidence regarding the effects of acute and/or chronic training stimulus on hematological parameters, such as hemoglobin (Hb), red blood cells (RBC), and hematocrit (Ht) [23]. It seems that there is a trend to observe increases in the above-mentioned hematological parameters after a period of soccer training, especially during the preparation phase [24]. The Hb, RBC, and Ht are important hematological parameters since they are linked to the player’s aerobic capacity, which is one of the physical aspects most trained during the pre-season [23,25]. In the case of biochemical parameters, they represent an important role for the monitoring of an athlete’s responses to the training loads imposed [26]. For instance, cortisol and testosterone levels represent good markers of training stress, with cortisol being associated to catabolic processes and testosterone to anabolic processes [27]. In fact, a study conducted on 25 soccer players affirmed that the high training volumes during the pre-season period causes a decrease in testosterone levels and an increase in cortisol levels [28]. Thus, in consequence of high training loads imposed, the athletes enter in a catabolic state that impairs physical performance [29].

These facts reinforce the need to be aware of other possible biochemical associations with the imposed training loads on athletes, especially during the pre-season period, where higher loads are imposed to athletes. Moreover, considering the injury rate during a soccer season, the neutrophils, monocytes, and eosinophils have an important role in the reaction to inflammation, acting as a defense through the process of phagocytosis. Lymphocytes and basophils also constitute a major importance in the immune system and in the defense against acute viral and bacterial infections [30], given that their relationships with training loads can be useful in relation to primary prevention of injuries. To the best of our knowledge, there is no study addressing different blood biomarkers variations and their interactions with different external load measures during the pre-season period. For those reasons, the purpose of this study is twofold: (i) Analyze the variations of chosen biological markers before and after the pre-season period and (ii) analyze the relationships between variations of biological markers and workload imposed on the players.

## 2. Materials and Methods

The article reported according to STROBE (the Strengthening the Reporting of Observational Studies in Epidemiology) guidelines for cohort designs [31].

### 2.1. Study Design and Setting

The present study followed an observational analytic cohort design with a quasi-experimental (pre-post) design. The period of data collection occurred between 2 June (beginning of the pre-season) and 19 September (after pre-season) of 2019. On 2 June and 19 September, players were assessed for their biological markers. Between the periods, the players were daily assessed for the training load parameters and wellbeing. From the blood samples collected to measure the biological markers, hematological and biochemical parameters were analyzed. All players were internally monitored in all training sessions during the pre-season period. All internal loads were monitored using subjective measures. For the quantification of subjective internal loads, the rate of perceived exertion (RPE) and the session-rate of perceived exertion (s-RPE) for general, breath, and neuromuscular perceived exertions were applied.

### 2.2. Participants and Study Size

Twenty-five professional soccer players (mean ± SD; age 28.1 ± 4.6 years old, height 176.7 ± 4.9 cm, body mass 72.0 ± 7.8 kg, and body fat percentage 10.3 ± 3.8%; body mass index using Quetelet equation: 23.4 kg/m^2^), from a professional club competing in the first league of Qatar (2019/2020 season), participated in this study. The inclusion criteria were (i) completed blood samples collections before and after pre-season period; (ii) no history of any neuropsychological impairments that could affect the results of the experiment (iii) absence of injuries, physical constraints, or illnesses during study period; (iv) absence of fatigue or illness during the blood samples collections of before and after the pre-season period; (v) participating in a minimum of 80% training sessions during the study period; and (vi) not have taken drugs such as pain killers or others that may influence the biochemical status during the two weeks before assessments. Technical staff and professional soccer players were informed regarding the study design and its related benefits and risks, as well as the main aims of the current investigation. All players signed an informed consent form to voluntarily participate in this study. All the professional soccer players in this study were treated according to the American Psychological Association (APA) guidelines, which ensure the anonymity of participants’ responses. The study protocol was approved by the Scientific Committee of School of Sport and Leisure (Melgaço, Portugal) with the code number CTC-ESDL-CE00118. The study followed the ethical standards of the Declaration of Helsinki.

### 2.3. Variables, Data Sources, and Quantitative Variables

#### 2.3.1. Anthropometry

Anthropometric measures were performed before and after the pre-season period, at the same time of the day. Body mass was measured using a body composition monitor (HD-351, Tanita, Arlington Heights, IL, USA) to the nearest 0.1 kg. While, the height was measured using a stadiometer to the nearest 0.1 cm (Seca 217, Ham- burg, Germany). Fat mass was also estimated using the body composition monitor. All measurements were performed by the same professional with a level 2 certification from the International Society for the Advancement of Kinanthropometry (ISAK). The experienced professional was considered mainly for the case of ensuring accuracy and precision in anthropometric measures related to height. Moreover, this professional also ensured the reproducibility conditions for the case of body composition analysis using bioimpedance. Those conditions were related to the protocol of cleaning the machine every time a player was measured, waiting the same time between players and after cleaning, and ensuring the same player’s position during the measurement.

#### 2.3.2. Biological Markers

##### Hematological Parameters

Laboratory blood samples were collected from players’ antecubital vein in a seated position. Blood samples (15 mL) were collected between 8:00 and 10:00 am, before and after the pre-season period. The blood samples were collected with all players in fasting, and with at least 12 h of rest (the time between the last training session, and the second blood draw) before the laboratory blood tests. All blood samples were centrifuged at 2500 rpm for 10 min, and the serum of each sample was immediately frozen at −80 °C for later biochemical analysis. Furthermore, 3 mL of blood were collected into vacutainer tubes containing ethylenediaminetetraacetic acid (EDTA). The blood samples were analyzed through flow cytometry, using a flow cytometer (FACSCalibur^TM^, BD Biosciences, San Jose, CA, USA) and using an automated hematology analyzer (Sysmex kx-21N Kobe, JAPAN). This method allowed to obtain hematological variables as follow: WBC: White blood cells; RBC: Red blood cells; Hb: Hemoglobin; Ht: Hematocrit; MCV: Mean corpuscular volume; MCHb: Mean corpuscular hemoglobin; MCHbC: Mean corpuscular hemoglobin concentration; RCDW: Red cells distribution width; PLT: Platelets; MPLTV: Mean platelets volume; NEUT: Neutrophils; LYMP: Lymphocytes; MNC: Monocytes; EOS: Eosinophils; BSO: Basophils; ANC: Absolute neutrophils count; AMC: Absolute monocytes count; ALC: Absolute lymphocytes count; and AEC: Absolute eosinophils count.

##### Biochemical Parameters

From the 15 mL of each blood sample, 7 mL of the original blood samples were placed into vacutainer tubes containing gelose for biochemical analysis. The blood serum was used to determine the following biochemical measures: Sodium, potassium, calcium, creatinine, alkaline phosphatase (ALP), albumin, ferritin, C-reactive protein (CRP), total cholesterol (TC), high-density lipoprotein cholesterol (C-HDL), low-density lipoprotein cholesterol (C-LDL), triglycerides (TG), cortisol, testosterone, and testosterone/cortisol ratio. All biochemical measures were analyzed using an Auto Chemistry Analyzer BM-100 (BioMaxima S.A., Lublin, Poland). The analyzer used was maintained by regular quality control procedures according to the manufacturer’s instruction to avoid any inconvenience during the procedures. The C-LDL was calculated based on the Friedewald Equation, i.e., TC-(TG/5)–C-HDL.

#### 2.3.3. Training Load Monitoring

##### Internal Loads

Regarding internal loads, subjective measures were used. The CR-10 scale was used to quantify each player rate of perceived exertion (RPE) [32]. Based on the CR-10 scale, a value of 1 means “very light activity” and a value of 10 means “maximal exertion”. Approximately 10 to 30 min after each training session, the RPE was individually collected and without the influence of others [33]. All players were familiarized with the RPE scale. Furthermore, to obtain the session-rate of perceived exertion (s-RPE), the RPE value attributed by each player was multiplied by the duration in minutes of each training session [34]. Thus, the s-RPE (expressed in arbitrary units [A.U.]), was used as the final outcome of subjective internal load measure to be analyzed in the present study. sRPE general, sRPE breath, and sRPE neuromuscular were also monitored as recommended elsewhere, for professional soccer players [19].

##### Well-Being Measures

For quantifying the well-being status of each player, a self-reported questionnaire comprised of a 7-point scale was used on a daily basis [15]. The questionnaire included questions involving stress, fatigue, delayed onset muscle soreness (DOMS), and sleep quality perceived levels. After the players answered the questions, the Hooper Index was used for analysis based on the scale, being calculated based on the sum of points from the four categories. This latter measure is the sum of the four question ratings. The questionnaire was sent to each player approximately 30-min before the training or match session.

##### Urine Color

The urine color chart [35] was implemented to the players before and after the pre-season period. At both times, urine was collected in a clear container and compared by the same observer with urine color chart. In this scale, the score varies between 1 (lightest) and 8 (darkest). The color “yellow”, “pale yellow”, or “straw yellow” indicates euhydration, while “dark” represents hypohydration [35]. The scale was previously confirmed as valid [36] to assess hydration.

#### 2.3.4. Statistical Procedures

Statistical analyses were carried out using the software Statistica (version 13.1; Statsoft, Inc., Tulsa, OK, USA). For all analyses, significance was accepted at *p* < 0.05. Descriptive statistics are represented as mean ± standard deviation (SD) with standard mean difference data. Tests of normal distribution and homogeneity (Kolmogorov–Smirnov and Levene’s, respectively) were conducted on all data before analysis. Paired sample *t*-test was used for determining differences as a repeated measures analysis (pre–post). Cohen d was the effect size indicator. To interpret the magnitude of the effect size, we adopted the following criteria: d = 0.20, small; d = 0.50, medium; and d = 0.80, large [37]. A Pearson’s correlation coefficient r was used to examine the relationship between the percentage of change of all biological mark [100 − (post × 100)/pre) and the training load (urine, sleep quality, stress, fatigue, soreness, Hooper Index, RPE general, RPE breath, RPE neuromuscular, sRPE general, sRPE breath, and sRPE neuromuscular [100 − (post × 100)/pre]). To interpret the magnitude of these correlations, we adopted the following criteria [37]: r ≤ 0.1, trivial; 0.1 < r ≤ 0.3, small; 0.3 < r ≤ 0.5, moderate; 0.5 < r ≤ 0.7, large; 0.7 < r ≤ 0.9, very large; and r > 0.9, almost perfect. Regression analysis was used to model the prediction of SMD blood biomarkers from remaining variables with positive correlation.

## 3. Results

First, a paired measure *t*-test with hematological parameters (WBC, RBC, RCDW, Hb, MCV, MCHb, MCHbC, MPLTV, EOS%, BASO%, NEUT%, LYMP%, MNC%, ALC, and AEC) showed no significant differences between before and after the pre-season period. There was a significant increase in PLT, while a significant decrease in AMC and ANC after the pre-season period (see Table 1, for more information).

A new paired measures *t*-test with biochemical parameters including, potassium, albumin, ferritin level, TC, TG, C-HDL, and, C-LDL, showed no significant differences between before and after the pre-season period. There was a significant increase in creatinine, ALP, CRP, cortisol, and testosterone, while a significant decrease in calcium and calcium corrected after the pre-season period (see Table 2, for more information).

**Figure 1:**
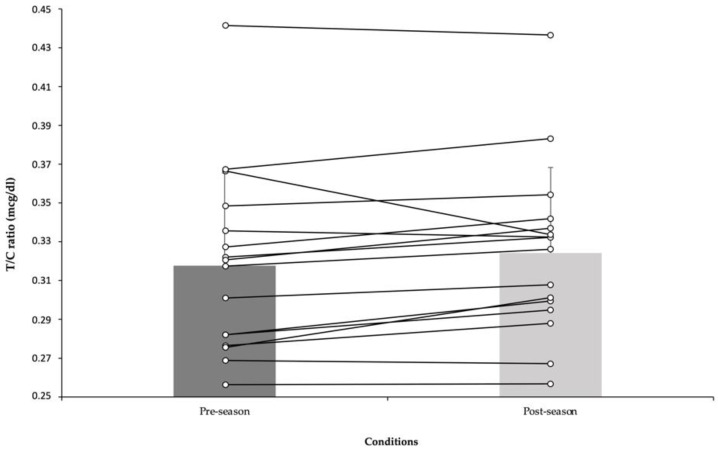
Before and after pre-season data (mean ± SD) of testosterone/cortisol ratio.

At this point, testosterone/cortisol ratio (T/C ratio) was calculated. In fact, the T/C ratio has been considered as an important physiologicaal variable to gauge individual condition and responses. In this sense, a *t*-test with data form the T/C ratio showed the same values before (0.317 ± 0.05) and after (0.324 ± 0.04) pre-season period [*t*(25) = 2.13, *p* = 0.07, d = 0]. Testosterone/cortisol ratio over the period can be found in Figure 1.

Regarding training load data, a paired measures *t*-test revealed no significant differences between before and after the pre-season period for urine color, stress, fatigue, sleep quality, and soreness measures. There was a significant increase in the Hooper Index, RPE (general), RPE (breath), RPE (neuromuscular), sRPE (general), sRPE (breath), and sRPE (neuromuscular) after pre-season compared to before pre-season (see Table 3, for more information).

Table 4 shows the relationships between percentage change of training load and the percentage of changes in hematological parameters. Very large positive correlations between RPE (general) and MCN% (*r* = 0.73; *p* = 0.001), and very large negative correlations between RPE (neuromuscular) and NEUT% (*r* = −0.71; *p* = 0.002) were found. Large positive correlations were found for the Hooper Index (*r* = 0.67; *p* = 0.004), soreness (*r* = 0.61; *p* = 0.01), and fatigue (*r* = 0.57; *p* = 0.02) with ALC percentage of changes. In addition, large positive correlations between sRPE (general) (*r* = 0.60; *p* = 0.012), RPE (neuromuscular) (*r* = 0.53; *p* = 0.03), and MNC percentage of changes were found. While, moderate negative correlations were found between stress and EOS (*r* = −0.50; *p* = 0.04) percentage of changes.

The associations between the percentage of change of training load and the percentage of changes of biochemical parameters can be seen in Table 5. Large negative correlations between sRPE (general) and sodium (*r* = −0.60; *p* = 0.013), between sleep quality (*r* = −0.58; *p* = 0.01), stress (*r* = −0.53; *p* = 0.033) and albumin, and between urine (*r* = −0.51; *p* = 0.04) and creatinine percentage of changes were found. On the other hand, large positive correlations between sRPE (breath) (*r* = 0.60; *p* = 0.014) and testosterone, and between RPE (general) (*r* = 0.56; *p* = 0.02) and C-HDL percentage of changes were found. While, moderate positive correlations between RPE (neuromuscular) (*r* = 0.50; *p* = 0.04) and ALP percentage of changes were found.

A multilinear regression analysis was performed to verify which variable of percentage of change of training load (agreement with the correlation analysis) could be used to better explain the percentage of change of hematological and/or biochemical parameters.

The percentage of change of urine color was a predictor of the percentage of change of creatine (*r* = −0.51). The percentage of change of sleep quality was a predictor of the percentage of change of albumin (*r* = 0.58). The percentage of change of stress was a predictor of the percentage of change of EOS and albumin (*r* = −0.50 and *r* = −0.53). The percentage of change of fatigue was a predictor of the percentage of change of ALC (*r* = −0.57). The percentage of change of soreness and hooper index were predictors of the percentage of change of ALC (*r* = 0.61 and *r* = 0.67), respectively. The percentage of change of RPE (general) was a predictor of the percentage of change of MNC and C-HDL (*r* = 0.73 and *r* = 0.56), respectively. The percentage of change of RPE (neuromuscular) was a predictor of the percentage of change of NEUT, MNC and ALP (*r* = −0.71, *r* = 0.53, and *r* = 0.50), respectively. The percentage of change of sRPE (general) was a predictor of the percentage of change of MNC and sodium (*r* = 0.60 and *r* = −0.60), respectively. The percentage of change of sRPE (breath) was a predictor variable of the percentage of change of testosterone (*r* = 0.60) (see Table 6. for more information).

## 4. Discussion

The purpose of this study was twofold: (i) Analyze the variations of biological markers before and after the pre-season period and (ii) analyze the relationships between variations of biological markers and workload imposed on the players. To the best of our knowledge, there is no study that addresses different blood biomarkers variations and their interactions with different internal load measures during the pre-season period. The major findings of the present study indicate that the Hooper Index, RPE (general, breath, and neuromuscular), and sRPE (general, breath, and neuromuscular) increased progressively after the pre-season. Likewise, PLT, creatinine, CRP, ALP, cortisol, and testosterone increased, whereas ANC, AMC, calcium, and calcium corrected decreased significantly after the pre-season period. Furthermore, several significant relationships were found between blood biomarkers, training load parameters (RPE and sRPE), and psychometric variables (the Hooper Index, fatigue, stress, soreness, and quality of sleep).

The pre-season is widely accepted to be the period with a high training load [38,39], and concomitant augmented risk of sustaining injuries [40]. High-quality pre-season soccer training plays a role not only in improving physical fitness (aerobic capacity), but also in injury prevention [41]. The monitoring of blood biomarkers before and after pre-season plays a role in increasing positive adaptation, and reducing the risk of injuries, illness, and overreaching caused by stress factors that occur during soccer matches over a season [42]. In our study, significant increases were found in training load parameters, the Hooper Index, RPE (general), RPE (breath), RPE (neuromuscular), sRPE (general), sRPE (breath), and sRPE (neuromuscular) after pre-season compared to before pre-season. Recent studies have frequently shown that internal or external workload indices [43]. In addition, the Hooper Index parameters were found to be higher during the pre-season period compared to other periods of the season [44,45]. The increase in training load parameters in the pre-season is usually due to the progressive overload principle of training, to prepare the players to meet the physical demands of the upcoming season [39].

Furthermore, the present study revealed a significant increase in PLT after the pre-season period. In the literature, there are studies with different results regarding the decrease [22], increase [46,47,48], or lack of changes [42] in PLT after long-term intensive soccer or different kinds of exercises. Michail et al. [46] revealed a similar conclusion to the results of the present study, as they found a significant increase from 231 × 103/µL to 244 × 103/µL of the PLT amount after the soccer intensive exercise intervention program. Moreover, a study conducted on 13 male soccer players, with significant augment in PLT levels (209.76 ± 33.83 to 249.76 ± 61.09 × 103/µL) was noted following 2 weeks of pre-tournament moderate-to-high intensity training period [49]. Contrary to our study, Ozen et al. [50] found an increase in PLT after the pre-season training period in well-trained young soccer players. However, their reported increase (pre: 205.57 ± 54.94, post: 214.85 ± 23.12) was not significant.

The reason for a high number of circulating PLT in the blood (thrombocytosis) after intense soccer exercise can be explained by epinephrine hormone secration, which has the ability to cause a strong contraction of the spleen (the storage area of one-third of the body’s PLT), and may play a role in the increase in PLT after exercise [51]. Likewise, it was declared that the mechanisms related to the increase in PLT after high intensity exercises were not clear [48]. However, those increases might be due to increased PLT production by cells in the bone marrow, and decreased removal of PLT from the blood, which was one of the functions of the spleen [48]. Another possible mechanism is shear and oxidative stress, which can activate PLT. Exercise-activated PLT contribute in growth factors liberation and proinflammatory mediators [52]. As in this study, an increase in PLT after intense exercise may also be associated with an improvement in performance. It was previously reported that hyperactive PLT have some pleiotropic effects on endurance sport performance, both by releasing ergogenic mediators and triggering an increase in performance-enhancing substances, such as nitric oxide into the circulation [53].

Regarding hematological parameters, our study revealed that the ANC and AMC significantly decreased after the pre-season period. Consistent with our findings, Heisterberg et al. [54] indicated that the numbers of circulating monocytes decreased at the end of a training season. In other study, it was noted that there was an increase in neutrophils and a decrease in lymphocytes after short periods of pre-tournament training [49]. Ozen et al. [50] reported no significant differences in subpopulations of leukocytes (lymphocytes, neutrophil, monocyte, and basophil percentage) after the pre-season period in young male football players. In a previous study, which was not consistent with the findings of our study in terms of neutrophil, an increase in neutrophil counts was found after regular and vigorous soccer exercises, and it was suggested that this situation was associated with minor inflammatory events [22]. In addition, contrary to our study, Dias et al. [55] notified an increase in total leukocyte, neutrophil, and monocyte counts, whereas lymphocytes reduced by the end of the season in volleyball athletes, and they also claimed that the increase in total neutrophils and monocytes might be due to muscle tissue remodelation, resulting from potential damage induced by training load and competition.

Furthermore, the present study revealed that decreases in ANC and AMC after the pre-season period may be related to the timing of blood collection after the last exercise session. In our study, there was a time of 12 h between the last training session, and the second blood draw (after the pre-season). This may have caused a short-term temporary suppression of the immune system in soccer players after the last training session, i.e., the previous day’s acute high-intensity exercise. This situation is defined as “open window” immunological phenomenon in the literature [56,57,58]. Moreover, previous studies showed that high-intensity exercises could lead to a short-term, acute inflammatory response [59,60,61,62]. Another study also supports the findings of the present study, in which the authors alleged that intense endurance activities decreased neutrophils, and monocytes in athletes, and this condition was related to the depression of the immune systems, which triggered an increased the risk of disease or infection, especially the pre-season period [63]. Lastly, regarding the leukocyte count and subpopulations in the pre-season period, the present study shows that there is no pathological condition, only the decreases in ANC and AMC may be associated with timing of blood collections. It can also be suggested that training in pre-season do not produce chronic effects on immune function and susceptibility to infection.

Creatinine is a metabolic product of a creatine breakdown during energy metabolism. The serum creatinine level is a known parameter for evaluating renal function in clinical medicine, and is used as an indicator of general health status and water-electrolyte balance in sports medicine [64]. The present study revealed that there was a significant increase in creatinine after the pre-season period. Our results are not consistent with some studies. For instance, Meyer & Meister [65] found only minor changes in creatinine levels in professional football players over a season. Another study revealed that there was no significant change in the serum creatinine level of rugby players before and after the training camp [66]. Furthermore, Andelković et al. [22] affirmed that serum creatinine levels in soccer players decreased significantly throughout the study, which might be related to the increase in training and competition workloads during the half competitive season. Prior studies on soccer player demonstrated that creatinine levels were higher in players with greater training and match loads (cumulative match-time) throughout the season [64], and also increased post-match in comparison with pre-match values due to the high intensity of the performance during the match [67]. Regarding creatinine as a by-product of muscle contraction, its rise after a match or higher training load, especially the pre-season period, could be due to the deterioration of muscle tissue [67]. Additionally, another study asserted that the increase in plasma creatinine after intense soccer exercise stemmed from the creatinine release from working muscles, dehydration, and/or reduction in renal blood flow and glomerular filtration rate [68]. In our study, there were negative large correlations between the percentage of change of urine color and percentage of change of creatinine. After creatinine is used by the muscles, it is filtered by the kidneys, and excreted in the urine, based on this information, this study reveals that urine is the determinant of the percentage change in creatinine.

Increased levels of oxidative stress are closely associated with markers of muscle damage with high inflammation [59]. CRP is the most common inflammation molecule of the body’s acute phase response, and it increases the inflammatory response to various stimuli that initiate the acute phase response [47,69,70]. In our study, significant increases were found in CRP values after post-pre-season compared to pre-pre-season. The CRP level has been found to increase during the inflammed state, that is, after intense exercise [71,72]. Significant increases in CRP after a soccer match in amateur soccer athletes was previously shown [47]. Mohr et al. [73] also found CRP values before (0.9 ± 0.1 mg/L), and after (1.3 ± 0.0 mg/L) the preparation period in professional soccer players. The studies mentioned above support the results of our study. However, these results differ from the study published by Radzimiński et al. [70], where it was found that elevated CRP values were not detected in soccer players during a pre-season sports camp (pre: 1.44 ± 0.7 mg/L, post: 0.83 ± 0.34 mg/L), i.e., above the reference range (<5.0 mg/L).

It was recently determined that decreases in CRP levels of futsal players, also asserted that reductions in the CRP level indicated that players adapt to the training load applied throughout the competitive season [59]. Radzimiński et al. [70], emphasized that inflammation in the bodies of pre-season soccer players might be the result of misuse of high-intensity training loads in a short time. On the other hand, a previous study stated that GPS variables associated with high-intensity activities, such as running speeds, accelerations, and decelerations were useful markers for detecting muscle damage or inflammation [74]. Similarly, Coppalle et al. [69] found a significant and very large correlation between total distance covered (>20 km/h) and CRP after the pre-season period in professional soccer players. The increment in CRP after the pre-season may be related to the frequent use of high-intensity activities in training during this period. However, the present study exhibited that this increase in CRP does not seem to reflect a pathological condition. Finally, it was pointed out that the rise in CRP after intensive exercise could be the result of mechanisms, such as the inflammatory response to injuries or agents (interleukin-6, i.e., the main stimulator of CRP secretion) that might be associated with elevated inflammation in athletes [47].

Moreover, the present study showed that alkaline phosphatase (ALP) significantly increased after the pre-season period. In the literature, some studies showed that ALP increased after intense soccer exercise [68,75], while some studies showed that no significant change in the ALP level of players with a higher training load over a season [64]. As in our study, the increase in ALP after intense soccer exercises might be associated with the result of some leakage from skeletal muscles of enzymes that play a role in the sustained release of ATP, and catabolize amino acids during exercises [68]. In addition, the increase in ALP after the pre-season period in our study may be explained by another study [76], as the authors suggested that the elevation in ALP levels reflected liver increased activity for gluconeogenesis, lipid peroxidation, and increased bone turnover triggered by the duration and intensity of exercise. Considering the CRP and ALP parameters related to inflammation, the physiological increase in CRP and ALP may be the result of acute high-intensity exercise [61] performed the day before blood collections. However, the present study demonstrated that pre-season intense soccer training does not cause any chronic effect on susceptibility to inflammation.

Calcium is a necessary mineral for proper growth, maintenance, and repair of bone tissue, nerve conduction, blood coagulation, and regulation of muscle contraction. Serum calcium level is tightly arranged by calcitonin and parathyroid hormone, independent of acute calcium intake [77,78,79]. In our study, statistically significant reductions were found in the calcium and calcium corrected after the pre-season period. The study of Mashiko et al. [66] does not coincide with our results, as they reported that there was no significant difference in the serum calcium level of rugby players after 20 days of pre-season intensive training. In our study, the decrease in calcium after an intense pre-season period can be explained as follows; calcium may leak into the tissue to create muscle contractions during exercise, so blood levels may decrease after intense exercises. In the report published by the UEFA expert group on nutrition in elite soccer, a daily calcium intake of 1300–1500 mg/dL is recommended for professional soccer players to optimize bone health in cases of relative energy deficiency in sports [77]. Accordingly, a recent study determined that soccer players did not meet their daily calcium needs in the pre-season period [79]. Given the importance of calcium for bone health, reductions in calcium concentration may result in decreases in bone mineral density, which can elevate the risk of injury to players throughout the season.

Cortisol and testosterone hormones play a role in catabolic and anabolic processes [80], are frequently used in studies as training stress markers, and these markers are closely associated with overreaching and overtraining syndromes [28,81]. The results found in the present study demonstrated that both cortisol and testosterone were significantly augmented in response to a soccer pre-season period. Di Luigi et al. [82] reported that salivary cortisol and testosterone level increased after an acute response to soccer exercise in young soccer player. Similarly, Muscella et al. [83] observed increases in both testosterone and cortisol levels after an intense training period in soccer referees. Nogueira et al. [84] remarked that testosterone increased, while the cortisol hormone decreased in futsal players after 4-weeks of pre-season. The same authors [84], noted that these results promoted an anabolic environment, which is also consistent with the finding of the study conducted by Perroni et al. [81]. Nevertheless, there are studies showing the formation of a catabolic environment (increases in cortisol, and decreases in testosterone levels) due to a high training load in the pre-season period [28,29,39,42]. It was reported that such a catabolic physiological environment could adversely affect various physical performance-related parameters such as speed, vertical jump height, and muscle strength throughout the season [28]. The T/C ratio is used to evaluate the balance between anabolic and catabolic activity [85,86], and represents a benefical tool in the early detection of overtraining [87]. The present study revealed that there were no significant changes in the T/C ratio after the pre-season training period. This result was supported by a previous study that showed that no significant changes in the T/C ratio after intense pre-season traninig in soccer players [88], and non-athletic men [83]. Contrary to our findings, recent studies observed significant reductions in the T/C ratio in response to a high volume of training sessions [39,89], and a period of congested match play [86,89] in professional soccer players. Similarly, another study demonstrated that a decrease equal or higher than 30% in the T/C ratio reflected state of catabolism, which resulted in a prolonged recovery time, fatigue, and deterioration of competitive soccer performance [90]. Additionally, our result was not similar to previous studies that reported that the T/C ratio increased significantly in team sports athletes after the pre-season period [81,84,85,89]. As in the present study, Botelho et al. [88] stated that a significantly unchanged T/C ratio after the pre-season period was associated with a favorable response to the training load, and adequate coping with training stresses. The current study revealed that the T/C ratio, which did not change significantly, and the conversely significant increases in cortisol and testosterone, after the pre-season could be explained by an environment that reflects a dynamic hemostatic balance between an anabolic and catabolic process in muscle [81,83]. This is very important in terms of both the prevention of the risk of injury of the players, and the quality of their physical performance during the training and competition season. Nonetheless, considering the testosterone, cortisol, and T/C ratio, the training load distribution and the load-rest relationship are well adjusted during the pre-season period, and the players have responded adequately to the training load without the accumulation of fatigue. Additionally, they probably have not experienced overreaching and overtraining. Moreover, the current study showed that the percentage of change of sRPE breath was a predictor variable of percentage of change of testosterone. Consistent with the present study, Peñailillo et al. [91] reported that the rate of perceived exertion was positively related to the change in testosterone levels. Accordingly, another study found that a higher internal training load (RPE-based) triggered anabolic stimulus (that is increases testosterone secretion) which positively affected performance in professional soccer players [92].

The present study indicated that negative large correlations were observed between a percentage of change of sleep quality and percentage of change of albumin, and also a percentage of sleep quality was a predictor variable of percentage of change of albumin. Sleep needs and rest are important for rapid recovery, and preventing the risk of illness, injury, and bad-overreaching in the pre-season period [93]. The deterioration in sleep quality due to a higher training load can be observed in the pre-season period, which may negatively affect biochemical parameters, especially albumin [30,42]. A previous study showed that that high-volume running exercises, which were frequently performed during pre-season training, caused a high sweating rate, which led to blood thickening, and as a result, it triggered an increase in the amount of albumin in the blood. Furthermore, in our study, blood measurements were performed in the morning hours (08.00–10.00 a.m.). Moreover, it was indicated that the augment in serum albumin levels in morning hours was closely related to the fact that normal blood thickening was not restored by overnight rest after exercise [66].

In the literature, there are limited studies examining the relationship between internal load indices (RPE, sRPE), wellness parameters (hooper index), and blood biomarkers. This is the first study to examine the relationship between pre-season training load (RPE, sRPE, and the Hooper Index) and blood biomarkers. Still, regarding the relationships between blood biomarkers, training load parameters (RPE and sRPE), and psychometric variables found in the present study, it is suggested that the internal load and Hooper Index parameters are associated with markers of inflammation and muscle damage. Interestingly, Dias et al. [55] reported that immune variables, such as total leukocytes, neutrophils, and lymphocytes might be modulated by training loads and by tactical and physical components. Indeed, Coppalle et al. [69] indicated that muscle damage or inflammation indicators, such as lactate dehydrogenase were correlated to RPE values, and suggested that the fatigue-related muscle damage enzyme increased at high perceived exertion levels. The same findings were also observed in our study. However, further research is needed to generalize the results from this study.

The present study contains some limitations that should be emphasized. First, the number of participants in our study was not very large. Considering the relationship between nutrition and hematological/biochemical parameters, no determination was made regarding the nutritional status of players in the pre-season period. In other words, the food consumption of players was not followed nor were there even supplements taken in the period. Furthermore, no measurements were made regarding the injury rate of the players. The relationship between pre-season training load parameters and injury rate could be examined. Despite the limitations mentioned above, the present study is to first examine different blood biomarkers variations and their interactions with different internal load measures during the pre-season period. In the future, by elevating the number of participants, it is recommended to increase the number of studies to compare blood biomarkers taking into account the gender and age factor in teams in different leagues according to player positions during the pre-season or the entire season, and to examine the relationships between these biomarkers, training load, and injury rate.

## 5. Conclusions

The present study revealed that intense training in the preseason period leads to decreases (ANC, AMC, calcium, and calcium corrected), and increases (PLT, creatinine, CRP, ALP, cortisol, and testosterone) in different hematological and biochemical markers. The present study also showed several significant relationships between blood biomarkers, training load parameters, and wellness variables. Given that, training load distribution is of critical importance in the optimization of blood biomarkers, especially during the pre-season period. In addition, ensuring a balance between the training load and blood biomarkers in the pre-season period contributes to the maintenance of high level physical performance of players during the entire season, and to prevent the risk of injury, bad-overreaching, and overtraining. Moreover, comprehensive monitoring of blood biomarkers in terms of hematological, nutritional, biochemical, muscle damage, and hormonal markers along with internal load indices and wellness measures can provide clearer insights into the mechanisms underlying players’ performance throughout the season.

## Figures and Tables

**Table 1:** Before and after pre-season data (mean ± SD) of anthropometric and hematological parameters (HP).

	Before Pre-season	After Preseason	% Change	*t*-Test (*p*)
Antropometric measures				
Body Mass (kg)	72.00 ± 6.37	69.92 ± 6.44	2.77	*p* = 0.001 ** |d = 0.32
Body Fat (%)	10.30 ± 3.15	8.10 ± 2.49	19.79	*p* = 0.001 ** |d = 0.77
Hematological parameteres				
WBC (10^9^/L)	5.38 ± 1.02	5.20 ± 1.21	−3.35	*p* = 0.10 |d = 0.16
RBC (10^12^/L)	4.94 ± 0.27	4.94 ± 0.34	0.00	*p* = 1.00 |d = 0.00
Hb (g/L)	14.63 ± 0.73	14.67 ± 0.65	0.27	*p* = 0.61 |d = −0.05
Ht (%)	43.03 ±1.44	42.93 ± 1.67	−0.23	*p* = 0.79 |d = 0.06
MCV (fL)	86.98 ± 4.58	87.64 ± 4.40	0.76	*p* = 0.34 |d = −0.14
MCHb (pg)	29.84 ± 1.58	29.92 ± 1.79	0.27	*p* = 0.26 |d = 0.16
MCHbC (g/dL)	33.91 ± 0.81	33.93 ± 0.85	0.06	*p* = 0.87 |d = −0.02
RCDW (%)	13.40 ± 0.83	13.49 ± 0.91	0.67	*p* = 0.28 |d = −0.10
PLT (10^3^/µL)	217.00 ± 38.41	230.94 ± 38.57	6.42	*p* = 0.006 * |d = −0.36
MPLTV (fL)	8.82 ± 0.90	8.68 ± 0.78	−1.59	*p* = 0.06 |d = 0.16
NEUT (%)	43.01 ± 9.83	42.99 ± 10.84	−0.05	*p* = 0.98 |d = 0.001
LYMP (%)	42.91 ± 9.55	44.36 ± 10.10	3.38	*p* = 0.24 |d = −0.14
MNC (%)	9.49 ± 1.97	9.04 ± 1.71	− 4.74	*p* = 0.09 |d = 0.24
EOS (%)	3.46 ±1.26	3.44 ±1.11	−0.58	*p* = 0.88 |d = 0.01
BASO (%)	0.70 ± 0.28	0.68 ± 0.26	−2.86	*p* = 0.35 |d = 0.07
ANC (10^9^/L)	2.26 ± 0.81	2.17 ± 0.79	−3.98	*p* = 0.006 * |d = 0.11
ALC (10^9^/L)	2.34 ± 0.50	2.26 ± 2.97	−3.42	*p* = 0.26 |d = 0.03
AMC (10^9^/L)	0.53 ± 0.11	0.44 ± 0.12	−16.98	*p* = 0.001 * |d = 0.78
AEC (10^9^/L)	0.19 ± 0.11	0.19 ± 0.11	0.00	*p* = 0.86|d = 0.00

HP: Hematological parameters; WBC: White blood cells; RBC: Red blood cells; Hb: Hemoglobin; Ht: Hematocrits; MCV: Mean corpuscular volume; MCHb: Mean corpuscular hemoglobin; MCHbC: Mean corpuscular hemoglobin concentration; RCDW: Red cell distribution width; PLT: Platelets; MPLTV: Mean platelets volume; NEUT: Neutrophils; LYMP: Lymphocytes; MNC: Monocytes; EOS: Eosinophils; BASO: Basophils; ANC: Absolute neutrophils count; ALC: Absolute lymphocytes count; AMC: Absolute monocytes count; AEC: Absolute eosinophils count; * Denotes significance at *p* < 0.05, and ** denotes significance at *p* < 0.01.

**Table 2:** Before and after pre-season data (mean ± SD) of biochemical parameters (BcP).

	Before Pre-Season	After Pre-Season	% Change	*t*-Test (*p*)
Sodium (mmol/L)	140.68 ± 1.22	140.76 ± 1.07	0.06	*p* = 0.70 |d = −0.06
Potassium (mmol/L)	4.01 ± 0.28	4.09 ± 0.35	2.00	*p* = 0.08 |d = −0.25
Creatinine (µmol/L);	83.55 ± 9.59	87.85 ± 8.75	5.15	*p* = 0.001 **|d = −0.46
Calcium (mmol/L)	2.29 ± 0.07	2.26 ± 0.05	−1.31	*p* = 0.007 ** |d = 0.49
Calcium Corr. (mmol/L)	2.29 ± 0.07	2.24 ± 0.05	−2.18	*p* = 0.015 * |d = 0.82
ALP (IU/L)	65.75 ± 11.40	75.13 ± 10.79	12.55	*p* = 0.001 **|d = −0.84
Albumin (g/L)	40.95 ± 2.53	40.41 ± 2.51	−1.32	*p* = 0.94 |d = 0.21
Ferritin (µg/L)	97.81 ± 59.15	101.69 ± 65.53	3.97	*p* = 0.16 |d = −0.06
CRP (mcg/mL)	2.64 ±0.55	3.04 ±0.63	15.15	*p* = 0.001 **|d = −0.67
TC (mmol/L)	4.35 ± 0.85	4.45 ± 0.69	2.30	*p* = 0.30 | d = −0.12
TG (mmol/L)	1.34 ± 0.96	1.23 ± 0.78	−8.21	*p* = 0.14 | d = 0.12
C-HDL (mmol/L)	1.27 ± 0.35	1.33 ± 0.38	4.72	*p* = 0.09 | d = −0.16
C-LDL (mmol/L)	2.70 ± 0.64	2.54 ± 0.61	−5.93	*p* = 0.24 | d = 0.25
Cortisol (mcg/dL)	20.72 ± 2.16	21.31 ± 1.95	2.85	*p* = 0.001 **|d = −0.28
Testosterone (mcg/dL)	6.51 ± 0.63	6.86 ± 0.69	5.38	*p* = 0.001 **|d = −0.52

BcP: Biochemical parameters; ALP: Alkaline phosphatase; CRP: C-reactive protein; TC: Total cholesterol; TG: Triglycerides; C-HDL: High-density lipoprotein cholesterol; C-LDL: Low-density lipoprotein cholesterol; * Denotes significance at *p* < 0.05, and ** denotes significance at *p* < 0.01.

**Table 3:** Before and after pre-season data of training loads (mean ± SD).

	Before Pre-Season	After Pre-Season	% Change	*t*-Test (*p*)
Urine color (A.U.)	2.43 ± 0.28	2.39 ± 0.20	−1.65	*p* = 0.65 | d = 0.16
Sleep Quality (A.U.)	2.75 ± 0.48	2.62 ± 0.39	−4.73	*p* = 0.30 | d = 0.29
Stress (A.U.)	2.33 ± 0.82	2.23 ± 0.31	−4.29	*p* = 0.58 | d = 0.16
Fatigue (A.U.)	2.88 ± 0.64	2.81 ± 0.62	−2.43	*p* = 0.64 | d = 0.11
Soreness (A.U.)	2.99 ± 0.66	2.74 ± 0.54	−8.36	*p* = 0.14 | d = 0.41
Hooper index (A.U.)	9.15 ± 1.63	10.36 ± 1.49	13.22	*p* = 0.01 * | d = 0.78
RPE (General) (A.U.)	3.23 ± 0.58	3.63 ± 0.44	12.38	*p* = 0.03 * | d = −0.77
RPE (Breath) (A.U.)	2.65 ± 0.28	3.11 ± 0.31	17.36	*p* = 0.006 ** | d = −1.55
RPE (Neuromuscular) (A.U.)	3.29 ± 0.50	3.05 ± 0.40	−7.29	*p* = 0.04 * | d = 0.53
sRPE (General) (A.U.)	217.81 ± 52.69	295.79 ± 46.84	35.80	*p* = 0.001 ** d = −1.56
sRPE (Breath) (A.U.)	179.46 ± 25.92	251.58 ± 31.89	40.19	*p* = 0.001 ** | d = −2.48
sRPE (Neuromuscular) (A.U.)	174.65 ± 44.05	287.65 ± 40.08	64.70	*p* = 0.001 ** | d = −2.56

RPE: Rate of perceived exertion; sRPE: Session rate of perceived exertion; A.U.: Arbitrary units * Denotes significance at *p* < 0.05, and ** denotes significance at *p* < 0.01.

**Table 4:** Pearson correlations between percentage change of HP (before and after the pre-season) and percentage change of training loads (before and after the pre-season).

	Urine	Sleep Quality	Stress	Fatigue	Soreness	Hooper Index	RPE (General)	RPE (Breath)	RPE (Neuromuscular)	sRPE (General)	sRPE (Breath)	sRPE (Neuromuscular)
WBC (10^9^/L)	*r* = 0.10 | *p* = 0.69	*r* = −0.03 |*p* = 0.90	*r* = 0.37 |*p* = 0.15	*r* = 0.28 |*p* = 0.28	*r* = 0.38 |*p* = 0.13	*r* = 0.37 |*p* = 0.15	*r* = 0.11 |*p* = 0.679	*r* = 0.2209 |*p* = 0.41	*r* = 0.05 |*p* = 0.83	*r* = 0.12|*p* = 0.65	*r* = 0.20 |*p* = 0.45	*r* = 0.26 |*p* = 0.32
RBC (10^12^/L)	*r* = 0.25 |*p* = 0.34	*r* = −0.33 |*p* = 0.20	*r* = −0.06 |*p* = 0.79	*r* = 0.03 |*p* = 0.89	*r* = 0.04 |*p* = 0.98	*r* = 0.07 |*p* = 0.78	*r* = 0.38 |*p* = 0.137	*r* = −0.2644 |*p* = 0.32	*r* = −0.08 |*p* = 0.75	*r* = 0.40 |*p* = 0.11	*r* = 0.02 |*p* = 0.99	*r* = −0.20 |*p* = 0.45
Hb (g/L)	*r* = 0.37 | *p* = 0.15	*r* = −0.24 |*p* = 0.35	*r* = −0.15| *p* = 0.55	*r* = −0.17|*p* = 0.52	*r* = −0.20|*p* = 0.45	*r* = −0.11|*p* = 0.67	*r* = 0.19 |*p* = 0.471	*r* = −0.4672 | *p* = 0.06	*r* = −0.28 | *p* = 0.28	*r* = 0.16 | *p* = 0.55	*r* = −0.26| *p* = 0.33	*r* = −0.20| *p* = 0.45
Ht (%)	*r* = 0.09 |*p* = 0.71	*r* = −0.20 |*p* = 0.45	*r* = −0.26 |*p* = 0.31	*r* = 0.03 |*p* = 0.89	*r* = −0.04 |*p* = 0.88	*r* = 0.01 |*p* = 0.96	*r* = 0.04 |*p* = 0.858	*r* = −0.3453 |*p* = 0.19	*r* = −0.28 |*p* = 0.29	*r* = 0.10 |*p* = 0.69	*r* = −0.17 |*p* = 0.52	*r* = −0.34 |*p* = 0.19
MCV (fL)	*r* = −0.20 |*p* = 0.44	*r* = 0.07 |*p* = 0.79	*r* = −0.05 |*p* = 0.82	*r* = 0.29 |*p* = 0.27	*r* = 0.25 |*p* = 0.34	*r* = 0.26 |*p* = 0.32	*r* = −0.15|*p* = 0.56	*r* = −0.08 |*p* = 0.75	*r* = −0.26 |*p* = 0.32	*r* = −0.10 |*p* = 0.71	*r* = −0.05 |*p* = 0.84	*r* = −0.24 |*p* = 0.36
MCHb (pg)	*r* = −0.16 |*p* = 0.54	*r* = 0.04 |*p* = 0.86	*r* = 0.03 |*p* = 0.90	*r* = −0.09|*p* = 0.73	*r* = −0.19 |*p* = 0.46	*r* = −0.25|*p* = 0.34	*r* = −0.03|*p* = 0.90	*r* = −0.11 |*p* = 0.68	*r* = −0.20|*p* = 0.43	*r* = −0.15 |*p* = 0.56	*r* = −0.23 |*p* = 0.38	*r* = −0.09 |*p* = 0.73
MCHbC (g/dL)	*r* = −0.20 |*p* = 0.45	*r* = −0.30 |*p* = 0.25	*r* = 0.01 |*p* = 0.96	*r* = −0.25|*p* = 0.33	*r* = −0.31 |*p* = 0.22	*r* = −0.40|*p* = 0.12	*r* = 0.12 |*p* = 0.63	*r* = 0.07 |*p* = 0.79	*r* = −0.18 |*p* = 0.4	*r* = −0.05 |*p* = 0.83	*r* = −0.18 |*p* = 0.49	*r* = −0.20 |*p* = 0.45
RCDW (%)	*r* = 0.02 |*p* = 0.92	*r* = −0.23 |*p* = 0.37	*r* = −0.28 |*p* = 0.28	*r* = −0.12|*p* = 0.64	*r* = −0.06 |*p* = 0.80	*r* = −0.16|*p* = 0.55	*r* = 0.15|*p* = 0.57	*r* = 0.23 |*p* = 0.39	*r* = −0.00 |*p* = 0.97	*r* = 0.07 |*p* = 0.77	*r* = 0.13 |*p* = 0.61	*r* = 0.11 |*p* = 0.67
PLT (10^3^/µL)	*r* = 0.14 |*p* = 0.60	*r* = 0.04 |*p* = 0.86	*r* = −0.02 |*p* = 0.93	*r* = −0.24|*p* = 0.35	*r* = −0.37|*p* = 0.15	*r* = −0.17|*p* = 0.52	*r* = −0.42|*p* = 0.10	*r* = −0.41 |*p* = 0.10	*r* = −0.36 |*p* = 0.16	*r* = −0.24 |*p* = 0.35	*r* = −0.31 |*p* = 0.22	*r* = 0.07 |*p* = 0.77
MPLTV (fL)	*r* = 0.06 |*p* = 0.81	*r* = −0.06 |*p* = 0.79	*r* = −0.03 |*p* = 0.90	*r* = 0.24 |*p* = 0.36	*r* = 0.23 |*p* = 0.38	*r* = 0.33 |*p* = 0.20	*r* = 0.05 |*p* = 0.83	*r* = −0.25 |*p* = 0.34	*r* = 0.13 |*p* = 0.61	*r* = 0.24 |*p* = 0.35	*r* = 0.12 |*p* = 0.64	*r* = −0.02 |*p* = 0.93
NEUT (%)	*r* = −0.17 |*p* = 0.52	*r* = −0.39 |*p* = 0.13	*r* = 0.12 |*p* = 0.64	*r* = −0.35|*p* = 0.17	*r* = −0.30 |*p* = 0.25	*r* = −0.25|*p* = 0.38	*r* = −0.04|*p* = 0.87	*r* = −0.39 |*p* = 0.13	*r* = −0.71 |*p* = 0.002 *	*r* = −0.11 |*p* = 0.66	*r* = −0.42 |*p* = 0.10	*r* = −0.02 |*p* = 0.93
LYMP (%)	*r* = 0.02 |*p* = 0.93	*r* = 0.14 |*p* = 0.60	*r* = 0.03 |*p* = 0.88	*r* = 0.03 |*p* = 0.90	*r* = 0.07 |*p* = 0.77	*r* = 0.07 |*p* = 0.79	*r* = 0.04 |*p* = 0.87	*r* = 0.34 |*p* = 0.19	*r* = 0.32 |*p* = 0.22	*r* = 0.03 |*p* = 0.90	*r* = 0.28 |*p* = 0.29	*r* = −0.03 |*p* = 0.89
MNC (%)	*r* = 0.09 |*p* = 0.73	*r* = −0.41 |*p* = 0.11	*r* = −0.21 |*p* = 0.43	*r* = −0.02|*p* = 0.93	*r* = −0.01 |*p* = 0.95	*r* = −0.16|*p* = 0.54	*r* = 0.73 |*p* = 0.001 *	*r* = 0.31 |*p* = 0.23	*r* = 0.53 |*p* = 0.03 *	*r* = 0.60 |*p* = 0.012 *	*r* = 0.37 |*p* = 0.15	*r* = 0.05 |*p* = 0.82
EOS (%)	*r* = 0.04 |*p* = 0.86	*r* = −0.30 |*p* = 0.25	*r* = −0.50 |*p* = 0.04 *	*r* = −0.08|*p* = 0.75	*r* = −0.04 |*p* = 0.87	*r* = −0.13|*p* = 0.60	*r* = 0.17 |*p* = 0.52	*r* = −0.20 |*p* = 0.44	*r* = −0.09 |*p* = 0.72	*r* = 0.16 |*p* = 0.54	*r* = −0.09 |*p* = 0.73	*r* = −0.21 |*p* = 0.43
BASO (%)	*r* = 0.12 |*p* = 0.63	*r* = −0.21 |*p* = 0.41	*r* = −0.13 |*p* = 0.63	*r* = 0.29 |*p* = 0.26	*r* = 0.26 |*p* = 0.32	*r* = 0.11 |*p* = 0.67	*r* = 0.47 |*p* = 0.06	*r* = 0.35 |*p* = 0.17	*r* = 0.29 |*p* = 0.26	*r* = 0.34 |*p* = 0.18	*r* = 0.29 |*p* = 0.27	*r* = −0.08 |*p* = 0.74
ANC (10^9^/L)	*r* = 0.08 |*p* = 0.76	*r* = −0.28 |*p* = 0.28	*r* = 0.18 |*p* = 0.48	*r* = −0.20|*p* = 0.44	*r* = −0.13 |*p* = 0.61	*r* = −0.02|*p* = 0.93	*r* = −0.07|*p* = 0.78	*r* = −0.40 |*p* = 0.12	*r* = −0.46 |*p* = 0.07	*r* = −0.01 |*p* = 0.96	*r* = −0.28 |*p* = 0.29	*r* = 0.06 |*p* = 0.81
ALC (10^9^/L)	*r* = −0.09 |*p* = 0.72	*r* = 0.36 |*p* = 0.17	*r* = 0.40 |*p* = 0.11	*r* = 0.57 |*p* = 0.02 *	*r* = 0.61 |*p* = 0.01 *	*r* = 0.67 |*p* = 0.004 *	*r* = 0.07 |*p* = 0.97	*r* = 0.33 |*p* = 0.20	*r* = 0.23 |*p* = 0.38	*r* = 0.13 |*p* = 0.62	*r* = 0.41 |*p* = 0.10	*r* = 0.03 |*p* = 0.89
AMC (10^9^/L)	*r* = 0.11 |*p* = 0.66	*r* = −0.13 |*p* = 0.62	*r* = −0.13 |*p* = 0.61	*r* = −0.25|*p* = 0.34	*r* = −0.15 |*p* = 0.57	*r* = −0.20|*p* = 0.45	*r* = 0.01 |*p* = 0.95	*r* = 0.04 |*p* = 0.86	*r* = 0.19 |*p* = 0.46	*r* = −0.06 |*p* = 0.79	*r* = −0.11 |*p* = 0.65	*r* = −0.16 |*p* = 0.53
AEC (10^9^/L)	*r* = 0.23 |*p* = 0.38	*r* = −0.23 |*p* = 0.37	*r* = −0.48 |*p* = 0.05	*r* = −0.21|*p* = 0.42	*r* = −0.20 |*p* = 0.43	*r* = −0.28|*p* = 0.29	*r* = 0.16 |*p* = 0.54	*r* = −0.10 |*p* = 0.69	*r* = 0.11 |*p* = 0.67	*r* = 0.18 |*p* = 0.48	*r* = −0.06 |*p* = 0.80	*r* = 0.06 |*p* = 0.80

HP: Hematological parameters; WBC: White blood cells; RBC: Red blood cells; Hb: Hemoglobin; Ht: Hematocrits; MCV: Mean corpuscular volume; MCHb: Mean corpuscular hemoglobin; MCHbC: Mean corpuscular hemoglobin concentration; RCDW: Red cell distribution width; PLT: Platelets; MPLTV: Mean platelets volume; NEUT: Neutrophils; LYMP: Lymphocytes; MNC: Monocytes; EOS: Eosinophils; BASO: Basophils; ANC: Absolute neutrophils count; ALC: Absolute lymphocytes count; AMC: Absolute monocytes count; AEC: Absolute eosinophils count; * Denotes significance at *p* < 0.05.

**Table 5:** Pearson correlations between percentage change of BcP (before and after the pre-seasonand percentage change of training loads (before and after the pre-season).

	Urine	Sleep Quality	Stress	Fatigue	Soreness	Hooper Index	RPE (General)	RPE (Breath)	RPE (Neuromuscular)	sRPE (General)	sRPE (Breath)	sRPE (Neuromuscular)
Sodium (mmol/L)	*r* = −0.17|*p* = 0.51	*r* = 0.08|*p* = 0.75	*r* = 0.16|*p* = 0.53	*r* = −0.31|*p* = 0.23	*r* = −0.35 |*p* = 0.18	*r* = −0.44|*p* = 0.08	*r* = −0.46|*p* = 0.07	*r* = −0.01|*p* = 0.94	*r* = 0.04|*p* = 0.86	*r* = −0.60|*p* = 0.013 *	*r* = −0.44|*p* = 0.08	*r* = −0.05|*p* = 0.98
Potassium (mmol/L)	*r* = −0.02 |*p* = 0.91	*r* = −0.35|*p* = 0.18	*r* = −0.38|*p* = 0.14	*r* = 0.06|*p* = 0.80	*r* = 0.05|*p* = 0.98	*r* = −0.11|*p* = 0.65	*r* = 0.38|*p* = 0.14	*r* = 0.04|*p* = 0.86	*r* = 0.40 |*p* = 0.12	*r* = 0.34|*p* = 0.18	*r* = 0.21|*p* = 0.43	*r* = −0.13|*p* = 0.62
Creatinine (µmol/L);	*r* = −0.51 |*p* = 0.04 *	*r* = 0.12|*p* = 0.65	*r* = 0.15|*p* = 0.55	*r* = −0.04|*p* = 0.99	*r* = −0.11|*p* = 0.67	*r* = −0.09|*p* = 0.73	*r* = 0.27|*p* = 0.30	*r* = 0.20|*p* = 0.43	*r* = 0.20|*p* = 0.45	*r* = 0.25|*p* = 0.34	*r* = 0.26|*p* = 0.32	*r* = 0.38|*p* = 0.14
Calcium (mmol/L)	*r* = 0.31 |*p* = 0.24	*r* = −0.43|*p* = 0.09	*r* = 0.05|*p* = 0.84	*r* = −0.29|*p* = 0.26	*r* = −0.23|*p* = 0.38	*r* = −0.07|*p* = 0.78	*r* = 0.14|*p* = 0.58	*r* = −0.45|*p* = 0.07	*r* = −0.48|*p* = 0.05	*r* = 0.24|*p* = 0.36	*r* = −0.20|*p* = 0.45	*r* = 0.13|*p* = 0.61
Calcium Corr. (mmol/L)	*r* = 0.06|*p* = 0.82	*r* = 0.10|*p* = 0.69	*r* = 0.36|*p* = 0.17	*r* = −0.02|*p* = 0.92	*r* = −0.06|*p* = 0.98	*r* = 0.27|*p* = 0.30	*r* = −0.19|*p* = 0.45	*r* = −0.46|*p* = 0.07	*r* = −0.38|*p* = 0.13	*r* = −0.02|*p* = 0.93	*r* = −0.19|*p* = 0.46	*r* = −0.05|*p* = 0.83
ALP (IU/L)	*r* = −0.22 |*p* = 0.40	*r* = −0.09|*p* = 0.71	*r* = −0.23|*p* = 0.376	*r* = −0.05|*p* = 0.98	*r* = −0.08|*p* = 0.76	*r* = −0.17|*p* = 0.52	*r* = 0.38|*p* = 0.14	*r* = 0.19|*p* = 0.47	*r* = 0.50|*p* = 0.04 *	*r* = 0.36|*p* = 0.17	*r* = 0.26|*p* = 0.31	*r* = 0.09|*p* = 0.72
Albumin (g/L)	*r* = 0.24 |*p* = 0.35	*r* = −0.58|*p* = 0.01 *	*r* = −0.53|*p* = 0.033 *	*r* = −0.25|*p* = 0.33	*r* = −0.17|*p* = 0.52	*r* = −0.22|*p* = 0.39	*r* = 0.38|*p* = 0.14	*r* = −0.15|*p* = 0.57	*r* = 0.13|*p* = 0.63	*r* = 0.37|*p* = 0.15	*r* = 0.03|*p* = 0.89	*r* = −0.07|*p* = 0.77
Ferritin (µg/L)	*r* = 0.39 |*p* = 0.13	*r* = 0.26|*p* = 0.32	*r* = 0.07|*p* = 0.796	*r* = 0.05|*p* = 0.84	*r* = 0.15|*p* = 0.56	*r* = 0.18|*p* = 0.49	*r* = −0.33|*p* = 0.20	*r* = −0.05|*p* = 0.83	*r* = 0.14|*p* = 0.58	*r* = −0.27|*p* = 0.30	*r* = −0.09|*p* = 0.73	*r* = −0.04|*p* = 0.85
CRP (mcg/mL)	*r* = 0.09 |*p* = 0.71	*r* = −0.22|*p* = 0.40	*r* = −0.19|*p* = 0.465	*r* = −0.05|*p* = 0.83	*r* = −0.04|*p* = 0.85	*r* = −0.19|*p* = 0.46	*r* = −0.18|*p* = 0.49	*r* = −0.10|*p* = 0.69	-.06|*p* = 0.80	*r* = −0.22|*p* = 0.40	*r* = −0.25|*p* = 0.33	*r* = −0.35|*p* = 0.17
TC (mmol/L)	*r* = 0.09 |*p* = 0.73	*r* = 0.23|*p* = 0.37	*r* = −0.15|*p* = 0.559	*r* = −0.12|*p* = 0.64	*r* = −0.16|*p* = 0.53	*r* = −0.09|*p* = 0.73	*r* = −0.06|*p* = 0.80	*r* = −0.13|*p* = 0.61	*r* = −0.21|*p* = 0.42	*r* = −0.09|*p* = 0.71	*r* = −0.15|*p* = 0.55	*r* = −0.31|*p* = 0.23
TG (mmol/L)	*r* = 0.26 |*p* = 0.31	*r* = 0.15|*p* = 0.56	*r* = −0.23|*p* = 0.377	*r* = 0.14|*p* = 0.58	*r* = 0.08|*p* = 0.75	*r* = 0.05|*p* = 0.84	*r* = −0.14|*p* = 0.59	*r* = 0.09|*p* = 0.71	*r* = 0.49|*p* = 0.05	*r* = −0.08|*p* = 0.76	*r* = 0.10|*p* = 0.68	*r* = −0.10|*p* = 0.69
C-HDL (mmol/L)	*r* = 0.11 |*p* = 0.68	*r* = −0.46|*p* = 0.07	*r* = −0.34|*p* = 0.195	*r* = 0.07|*p* = 0.79	*r* = 0.06|*p* = 0.81	*r* = −0.08|*p* = 0.75	*r* = 0.56|*p* = 0.02 *	*r* = 0.21|*p* = 0.43	*r* = 0.12|*p* = 0.65	*r* = 0.4135|*p* = 0.111	*r* = 0.24|*p* = 0.35	*r* = −0.44|*p* = 0.08
C-LDL (mmol/L)	*r* = 0.06 |*p* = 0.82	*r* = 0.1752|*p* = 0.516	*r* = 0.050|*p* = 0.854	*r* = −0.03|*p* = 0.88	*r* = −0.12|*p* = 0.63	*r* = −0.08|*p* = 0.74	*r* = 0.14|*p* = 0.58	*r* = 0.14|*p* = 0.59	*r* = 0.07|*p* = 0.97	*r* = 0.0588|*p* = 0.829	*r* = 0.05|*p* = 0.85	*r* = −0.04|*p* = 0.86
Cortisol (mcg/dL)	*r* = 0.32 |*p* = 0.21	*r* = 0.2735|*p* = 0.305	*r* = −0.06|*p* = 0.812	*r* = 0.14|*p* = 0.60	*r* = 0.14|*p* = 0.59	*r* = 0.25|*p* = 0.34	*r* = −0.43|*p* = 0.09	*r* = −0.21|*p* = 0.41	*r* = 0.04|*p* = 0.87	*r* = −0.2787|*p* = 0.296	*r* = −0.07|*p* = 0.77	*r* = −0.04|*p* = 0.86
Testosterone (mcg/dL)	*r* = −0.18 |*p* = 0.49	*r* = −0.2311|*p* = 0.389	*r* = 0.013|*p* = 0.959	*r* = 0.41|*p* = 0.11	*r* = 0.47|*p* = 0.06	*r* = 0.43|*p* = 0.09	*r* = 0.25|*p* = 0.34	*r* = 0.46|*p* = 0.07	*r* = 0.10|*p* = 0.69	*r* = 0.3913|*p* = 0.134	*r* = 0.60|*p* = 0.014 *	*r* = 0.05|*p* = 0.84

BcP: Biochemical parameters; ALP: Alkaline phosphatase; CRP: C-reactive protein; TC: Total cholesterol; TG: Triglycerides; C-HDL: High-density lipoprotein cholesterol; C-LDL: Low-density lipoprotein cholesterol; * Denotes significance at *p* < 0.05.

**Table 6:** Regression analysis for the percentage change of training loads based on percentage change on the remaining blood biomarkers.

Training Load	Biomarkers	b *	SE of B *	R^2^	Adjusted R^2^	F	*p*
% change of urine color	% change of creatine	−0.51	0.22	0.26	0.21	5.10	0.04
% change of sleep quality	% change of albumin	−0.58	0.21	0.34	0.30	7.44	0.01
% change of stress	% change of EOS	−0.50	0.23	0.25	0.20	4.86	0.04
	% change of albumin	−0.53	0.22	0.28	0.23	5.63	0.03
% change of fatigue	% change of ALC	0.57	0.21	0.32	0.27	6.80	0.02
% change of soreness	% change of ALC	0.61	0.21	0.37	0.33	8.40	0.01
% change of hooper index	% change of ALC	0.67	0.19	0.45	0.41	11.57	0.004
% change of RPE general	% change of MNC	0.73	0.18	0.53	0.50	16.02	0.001
	% change of C-HDL	0.56	0.21	0.32	0.27	6.70.	0.02
% change of RPE neuromuscular	% change of NEUT	−0.71	0.18	0.50	0.47	14.41	0.001
	% change of MNC	0.53	0.22	0.28	0.23	5.49	0.03
	% change of ALP	0.50	0.23	0.25	0.19	4.72	0.04
% change of sRPE general	% change of MNC	0.60	0.21	0.37	0.32	8.28	0.01
	% change of sodium	−0.60	0.21	0.36	0.31	8.05	0.01
% change of sRPE breath	% change of testosterone	0.60	0.21	0.36	0.31	7.90	0.01

* Denotes significance at *p* < 0.05.
